# Molecular Bases of Alzheimer’s Disease and Neurodegeneration: The Role of Neuroglia

**DOI:** 10.14336/AD.2018.0201

**Published:** 2018-12-04

**Authors:** Antonina Luca, Carmela Calandra, Maria Luca

**Affiliations:** ^1^Department of Medical and Surgical Sciences and Advanced Technologies “G.F. Ingrassia”, University Hospital Policlinico-Vittorio Emanuele, Catania, 95100 Sicily, Italy; ^2^Department of General Surgery and Medical-Surgical Specialties, Dermatology Clinic, University Hospital Policlinico-Vittorio Emanuele, Catania, 95100 Sicily, Italy

**Keywords:** neuroglia, Alzheimer’s Disease, neurodegeneration, oxidative stress, neuroinflammation, glycogen synthase kinase 3

## Abstract

Neuroglia is an umbrella term indicating different cellular types that play a pivotal role in the brain, being involved in its development and functional homeostasis. Glial cells are becoming the focus of recent researches pertaining the pathogenesis of neurodegenerative disorders, Alzheimer’s Disease (AD) in particular. In fact, activated microglia is the main determinant of neuroinflammation, contributing to neurodegeneration. In addition, the oxidative insult occurring during pathological brain aging can activate glial cells that, in turn, can favor the production of free radicals. Moreover, the recent Glycogen Synthase Kinase 3 (GSK-3) hypothesis of AD suggests that GSK3, involved in the regulation of glial cells functioning, could exert a role in amyloid deposition and tau hyper-phosphorylation. In this review, we briefly describe the main physiological functions of the glial cells and discuss the link between neuroglia and the most studied molecular bases of AD. In addition, we dedicate a section to the glial changes occurring in AD, with particular attention to their role in terms of neurodegeneration. In the light of the literature data, neuroglia could play a fundamental role in AD pathogenesis and progression. Further studies are needed to shed light on this topic.

Neuroglia is an umbrella term indicating different cellular types exerting several functions in the physiology and pathology of the central (CNS) and peripheral nervous systems. Initially defined as a “connective tissue that binds nervous elements together” [1], neuroglia plays a pivotal role in the brain, being involved in its development and functional homeostasis [2]. A recently published genetic study concludes that “glial-specific genes predict age with greater precision than neuron-specific genes” [3]; hence, the involvement of glial cells in the healthy as well as in the pathological brain could be even more surprising than we currently know. Glial cells are becoming the focus of recent researches pertaining the pathogenesis of neurodegenerative disorders, Alzheimer’s Disease (AD) in particular. In fact, activated microglia is the main determinant of neuroinflammation, contributing to neurodegeneration [4]. In addition, the oxidative insult occurring during pathological brain aging can activate glial cells that, in turn, can favor the production of free radicals [5]. Moreover, the recent Glycogen Synthase Kinase 3 (GSK-3) hypothesis of AD suggests that GSK3, involved in the regulation of glial cells functioning, could exert a role in amyloid deposition and tau (a microtubule-associated protein) hyper-phosphorylation [6-8]. Improving the knowledge regarding the role of neuroglia in AD would be useful to clarify the complex pathogenesis of this disorder. In fact, the functions of glial cells in the healthy and pathological brain are extremely various and complex and, in some cases, still debated. In this review, we briefly describe the main physiological functions of the glial cells and discuss the link between neuroglia and the most studied molecular bases of AD. More specifically, this review is articulated into three sections, discussing the following topics: 1) Physiological functions of neuroglial cells; 2) Molecular bases of AD (namely oxidative stress, neuroinflammation and dysfunctional GSK-3 pathway) and involvement of neuroglia; 3) Role of the glial changes occurring in AD in terms of neurodegeneration.

## Neuroglial cells

Glial cells outnumber the neurons and are involved in a variety of physiological functions [2]. CNS neuroglia consists of microglia, representing myeloid-lineage cells, and macroglia, including different cell types sharing an ectodermal origin: astrocytes, oligodendrocytes, ependymal cells, neural/glial antigen 2 proteoglycan (NG2)-positive cells and radial glial cells. The latters disappear in the adult brain but generate a progeny of adult neural stem cells [9,10,2]. Glial cells exert roles of primary importance for the CNS, being involved in the formation of myelin sheaths around axons, in the generation and functioning of synapses, as well as in the maintenance of physiological concentration of ions and neurotrasmitters [2]. The specific functions of the CNS glial cells are described below.

## Functions of macroglial cells

### Astrocytes

Astrocytes display different functional specializations and are involved in a great number of physiological processes. They differ in terms of distribution and functioning according to brain regions, morphology, target expression, Ca^2+^ signals, brain circuits and microcircuits [11]. As described below, if on the one hand astrocytes play a central role in the cerebral homeostasis, on the other hand they can contribute to neurodegeneration [12]. During development, they contribute to the formation of neurons and neuronal circuits [13]; in the adult brain, they engage in a close interaction with neurons, forming the so-called tripartite synapsis. This term refers to the active role of astrocytes in synaptic transmission and plasticity, no more considered to rely on the exclusive crosstalk between pre and post synaptic neurons. The release of neurotransmitters by presynaptic terminals results in the activation, through a Ca^2+^-dependent mechanism, of astrocytes, that are in turn able to release neurotransmitters that regulate both postsynaptic and presynaptic terminals, thus modulating the cellular excitability [14,15]. A fascinating example lays in the fact that when neurons release glutamate, the major excitatory neurotransmitter of the CNS, astrocytes are stimulated to liberate the same neurotransmitter. The glutamatergic signaling provides a feedback to the neurons, guaranteeing a bidirectional communication. In addition, in order to avoid neuronal damage due to excessive excitability, astrocytes remove glutamate at synaptic level [16]. In addition, along with microvessels and neurons, astrocytes form the so-called neurovascular unit and participate in the regulation of both blood brain barrier (BBB) and brain microcirculation [17-19]. Moreover, astrocytes are involved in the energy sourcing for the brain, transporting substances from the bloodstream and sharing their metabolism with neurons, for example releasing lactate in the extracellular space to be up-taken by them [20,21]. Astrocytes also represent the main regulators of manganese concentrations in the brain. In cellular models of manganese neurotoxicity, activated astrocytes express several inflammatory cytokines and trigger a complex inflammatory cascade whose final step is neuronal apoptosis [22]. In addition, astrocytes express class II major histocompatibility complex (MHC) antigens and costimulatory molecules, thus contributing to the brain immune response [23]. Expressing neurotrophic factors, such as brain-derived neurotrophic factor (BDNF), astrocytes contribute to cerebral trophism [24].

### Oligodendrocytes

The main function of olygodendrocytes is the formation and maintenance of the myelin sheath around the axons, fundamental for the rapid saltatory propagation. The myelination process requires a unique metabolism: olygodendrocites consume high amounts of oxygen and ATP and produce free radicals, exposing themselves to the risk of damage. In fact, oligodendrocytes represent highly vulnerable glial cells, since not only the myelination process, but also the steps leading to their migration and differentiation are extremely delicate and strictly programmed [25]. In addition, oligodendrocytes provide long-term axonal support and their metabolism is fundamental for a healthy brain; in fact, alterations in their lipid metabolism have been related to neurodegenerative disorders characterized by demyelination, such as multiple sclerosis [26]. Oligodendrocytes and the axons they myelinate represent intertwined functional units. During development, neurotransmitters released by axons stimulate the differentiation of oligodendrocytes progenitors in myelinating cells. In adulthood, neurotrasmitters released by axons and astrocytes affect oligodendrocytes signaling [27]. Oligodendrocytes also communicate with microglia, since they secrete the exosomes, small membrane vescicles that are internalized by microglial cells in order to guarantee the turnover of the oligodendrocytes membrane without activating an inflammatory response. This mechanism is also part of the neurons-oligodendrocytes relationship, since exosomes are internalized by neurons, too [28]. However, oligodendrocytes could potentially express autoantigens; therefore, this peculiar mechanism could trigger the autoimmune processes leading to demyelination, via the stimulation of a dangerous subset of microglial cells [29]. The death of oligodendrocytes is in fact sufficient to activate autoimmune responses against myelin [30]. On the other hand, oligodendrocytes modulate the immune functions of the CNS, expressing MHC class I molecules and immune receptors, as well as regulating microglia through the expression of a variety of cytokines and chemokines [31]. Oligodendrocytes also express neurotrophic factors, such as nerve growth factor and BDNF, to support neurons [32].

### Ependymal cells

Ependymal cells are epithelial, multi-ciliated cells located in the ventricular system [33]. In the early development, they could represent an axonal guidance system. They are believed to derive from radial glia progenitor cells losing their primary (not-motile) cilia and acquiring secondary, motile ones. According to their location along the ventricles, they acquire their cilia at specific moments during development [34,33]. However, different subtypes of ependymal cells, displaying structural differences, exist. For example, an uniciliated subtype has been demonstrated in the third ventricle floor [35]. Ependymal cells participate to the creation of the BBB and the blood-cerebrospinal fluid barrier (BCB). In particular, within the ventricular system, the choroid plexus (CP) is characterized by specialized ependymal cells expressing aquaporins, up-taking water from blood to ventricles for the cerebrospinal fluid (CSF) formation [36]. Both in developing and mature brain, the directional flow of the CSF is guaranteed by the cooperation between ciliated cells, finely regulated by several neurotransmitters [34,37]. The disruption of the ependymal system is involved in hydrocephalus formation as well as immune-related brain damages, such as the massive cellular infiltration occurring during infections [36]. In a model of murine neurocysticercosis, it has been proposed that leukocytes could extravasate from the pial vessels and then pass through the ependymal cells. The cellular infiltration occurring during this infection has been related to the alterations affecting the junctional complex proteins of activated ependyma adjacent to the internal leptomeninges [38]. Both in physiological and pathological conditions, the maintenance of a proper ependymal layer is guaranteed by mechanisms of repair (probably stimulated by purinergic signaling) performed by astrocytes [39]. Similarly to the other glial cells, ependymal cells express neurotrophic factors [40]. Their role as stem cells in the adult brain is still controversial, but some evidences suggest that they lack proliferative properties once mature [41].

### Radial glial cells and NG2-positive cells

Radial glial cells and NG2-positive cells could be defined as multi-potent stem cells representing the progenitors of mature cells [42,43]. Almost all the cells in the developing brain are produced in two germinal zones near to the ventricular walls: the ventricular zone (VZ) and subventricular zone (SVZ). The VZ consists of an epithelium rich in multi-potent neural stem cells (primary progenitors), while the SVZ is rich of secondary progenitors derived from the VZ. The VZ was thought to disappear and give rise to the non-differentiating ependymal layer, but the presence of stem-like cells has been demonstrated along the ventricular walls. Moreover, a VZ persisting in the walls of the neonatal lateral ventricle between the lumen and the SVZ has been demonstrated [44]. The fact that brain stem cells can give rise to cellular types belonging to both neuronal and glial lineages, also according to where they are located, undermines the theory that neurogenesis and gliogenesis are two distinct and independent processes [45,46]. In adulthood, the SVZ and the hippocampal dentate gyrus represent the “headquarters” of the brain stem cells [47]. As opposed to the original thought that mature brain was unable to activate neurogenesis, it is now recognized that it can initiate mechanisms of restoration, thanks to glial cells maintaining their ability to differentiate as well as activated glia re-acquiring neurogenic potential [48]. The importance of stem cells, such as radial glial cells, could not be limited to brain development, since the link between progenitors and mature cells could go beyond what expected [42]. The name radial glia is due to the peculiar feature of these cells, showing both characteristics of neuroepithelial cells (radial processes) and of astrocytes in particular (content of glycogen granules and expression of molecules such as the astrocyte-specific glutamate transporter) [49]. In fact, even if they can differentiate into neurons and ependymal cells, they are mostly considered astrocytes progenitors [49,34]. While in some species radial glial cells are still present once the brain development is completed, in adult mammals they disappear after maturing into astrocytes. A subgroup of astrocytes, called type B cells, maintains the ability to differentiate into neurons, thus acting as stem cells and contributing to neurogenesis even in adulthood. They have been detected in the subventricular zone and in the hippocampus [50,48,51], that contain NG2-positive cells also [52]. As opposed to radial glial cells, NG2-positive cells persist in the adult brain. They are characterized by irregular cell bodies and multiple fine branches and express proteoglycan NG2 (hence their name) [53,54]. They do not show characteristics typical of neurons, mature oligodendrocytes, astrocytes or even microglia [43,53]. In vitro, they differentiate into oligodendrocytes, so they are considered to be their progenitors. They could contribute to re-myelination after injury, in fact they concentrate in demyelinating lesions [43]. In addition, since they receive synaptic input from neurons, they could be involved in the neuronal physiology [53]. They seem to be able to differentiate into oligodendrocytes and astrocytes, too, even if their role as multipotent neural stem cells is still controversial [55,56]. In vitro experiments in mice suggest a role of NG2-positive cells as a cellular reservoir for the generation of inhibitory interneurons in the postnatal hippocampus [57].

### Functions of microglia

Microglial cells are characterized by many branching processes and little cytoplasm. They express a peculiar pattern of K+ channels, different from that of neurons and other glial cells [58]. They contribute to the brain development, regulating programmed cell death, synapse maturation and pruning, the latter being the process of synapse elimination occurring between early childhood and puberty [59]. They are also responsible for the elimination of dysfunctional synapses (synaptic stripping) and they stimulate adult neurogenesis [60]. Microglial cells are the first cellular types to appear in the developing brain, but their maturation is fully achieved later in development, when astrocytes appear. In fact, these glial cells are closely related and co-operate in several processes, such as angiogenesis, synaptogenesis, axonal outgrowth and synaptic pruning [61]. For example, astrocytes can stimulate the elimination of synapses by microglia through the release of growth factors. In adulthood, the cross-talk between microglia, neurons and astrocytes continues. It is fascinating how microglia can perceive neuronal activity, also thanks to their glutamate receptors, as well as astrocyte activity, in the form of Ca^2+^ waves [58]. In animal models, it has been demonstrated that microglial cells are dynamically involved in the regulation of the extracellular environment under both physiological and pathological conditions [62]. Most importantly, microglial cells are the main actors of both innate and adaptive immune response of the CNS [9]. Various stimuli can induce microglial activation, such as infections, trauma, ischemic and neurodegenerative lesions. Activated microglia switches from a ramified state to an ameboid one, resembling macrophages. In fact, microglial cells are called resident brain macrophages. In case of acute injury, they migrate to the site of damage [63]. They perform immune functions not only exerting pinocytic activity, but also being directly involved in the antigen presentation [64,65]. Microglia can assume diverse reactive states, also under the differential stimulation by neurotransmitters (such as glutamate and γ-amino butyric acid) regulating the release of microglial cytokines [58,66]. Under physiological conditions, microglia release controlled levels of pro-inflammatory cytokines, such as tumor necrosis factor alpha (TNF-α), since inflammation is involved in the regulation of synaptic connectivity and adult neurogenesis. In response to injury, the acute neuroinflammation induced by activated microglia represents a defensive mechanism, aimed to eliminate the cause of injury, contain the damage and initiate the mechanisms of repair. Alternatively, the damaged cells are directed towards apoptosis. For example, in the previously reported model of manganese neurotoxicity, the initiation of the neuroinflammatory mechanisms in astrocytes has been demonstrated to depend on microglia: without microglial cells, astrocytes could not induce apoptosis. It is now known that activated microglia can differentiate into two distinct macrophage subtypes: M1 or M2. The M1 phenotype determines a pro-inflammatory condition, with the release of inflammatory cytokines and oxidative compounds, while the M2 phenotype promotes tissue repair favoring the release of anti-inflammatory cytokines and neurotrophic factors [22]. Chronic neuroinflammation, with the sustained release of massive levels of pro-inflammatory compounds, represents a crucial step towards neurodegeneration, as described below [67,60,68].

### Molecular bases of AD: the role of neuroglia

AD is a neurodegenerative disorder characterized by a progressive impairment of the cognitive performances (e.g. memory, speech, executive functions) frequently associated with behavioral disturbances [69]. In terms of anatomical features, AD is characterized by the presence, in the brain, of senile plaques and neurofibrillary tangles (NFTs). Senile plaques are extracellular deposits of amyloid plaques, mostly composed of amyloid beta (Aβ) 40 or 42, derived from the proteolytic cleavage of amyloid precursor protein (APP) by beta and gamma secretases. They accumulate in the brain tissue and in the vessels, leading to the so-called cerebral amyloid angiopathy. NFTs are intracellular protein depositions of hyper-phosphorylated tau protein, which filaments accumulate in dystrophic neurites [70-72]. These lesions are accompanied by activated microglia as well as loss of neurons, synapses and neuroglial cells [73,74]. According to the amyloid cascade hypothesis of AD, the crucial step towards neurodegeneration is the deposition of insoluble Aβ in plaques in the brain tissue: the hyper-phosphorylation of tau, the formation of NFTs and, more generally, the disease progression is secondary to amyloid deposition, due to the imbalance between amyloid production and clearance [75]. Familial forms of AD have been linked to mutations in the genes of presenilin 1, presenilin 2 and amyloid precursor protein, affecting APP processing [71]. Considering sporadic AD, the most frequent type of AD, the deposition of Aβ in the form of senile plaques is higher in individuals carrying the ε4 allele of the apolipoprotein E (ApoE), a major cholesterol carrier mostly produced by astrocytes that favors Aβ deposition in the brain and vessels [70]. From a pathophysiological point of view, as detailed in the following paragraphs, the AD brain is characterized by protracted neuroinflammation, mostly due to activated microglia, synaptic loss, largely related to astrogliosis, as well as a myelin breakdown sustained by vulnerable oligodendrocytes. In this context, the impaired CSF dynamic, due to dysfunctional ependymal cells, leads to an altered amyloid clearance, thus favoring Aβ toxicity. Moreover, the cellular loss is not successfully counterbalanced by the brain stem cells through efficient neurogenesis and gliogenesis. The complex pathogenesis of AD could be, at least in part, re-conducted to the following intertwined molecular bases: oxidative stress (OS), neuroinflammation, dysfunctional GSK-3 pathway. When examining them, the involvement of neuroglia stands out as a common element that confirms the importance of glial cells in both the physiology and pathology of the brain.

### Oxidative stress

OS occurs when the balance between the production and elimination of reactive oxygen species (ROS) fails. ROS are mostly generated from mitochondria and represent by-products of cellular metabolism. They are involved in several physiological processes, such as apoptosis, signaling and cellular response to stress. However, since they are highly reactive species, they are potentially dangerous. The anti-oxidative defense system consists of non-enzymatic (e.g. vitamins) and enzymatic (e.g. superoxide dismutase) antioxidants [76]. Since it is universally recognized that OS and aging are strictly linked, and AD is an age-related illness, many researchers have focused their attention on the role of OS in AD. In normal aging, the production of ROS increases, while the antioxidant defense system is impaired, so that fundamental biomolecules, such as nucleic acids, lipids, proteins or carbohydrates are at risk of OS-related damages [77]. Consuming a lot of oxygen, the brain is exposed to the accumulation of ROS; however, it does not have a powerful antioxidant defense system. Being rich in fatty acids, it is susceptible to lipid peroxidation, that has been clinically related to the frontal cognitive impairment, since the frontal lobe (fundamental for both cognitive and executive functions) is highly sensitive to OS [78,79]. In the aged brain, the OS-related damage to the polyunsatured fatty acids, already depleted in the elderly, negatively affects the fluidity of the neuronal membrane [76,80]. Despite the role of mitochondrial DNA (mtDNA) mutations in aging has been questioned, the aged brain shows OS-related mtDNA alterations that increase the production of ROS; as a result, OS can further damage mitochondria and other fundamental cellular components. ROS are also responsible for an accelerated telomere shortening, thus anticipating the cellular loss [76,81].

### Oxidative stress in AD

As far as pathological brain aging is considered, brains affected by AD display molecular features typical of OS-related injuries: protein oxidation, lipid peroxidation, damaged DNA and accumulation of metals that, showing a catalytic activity, are great producers of ROS [82]. Markers of OS have been demonstrated in AD patients from the very early stages of neurodegeneration. The oxidative damage seems to affect the lipid fraction in particular, as demonstrated by the high levels of markers of lipid peroxidation, such as malondialdehyde and 4-hydroxynonenal, in plasma/serum of AD patients compared to controls. Conversely, AD patients are characterized by a considerable impairment of the non-enzymatic antioxidant defense system (e.g. vitamins A and E) [83]. The overproduction of ROS in AD could be primarily due to the functional alterations affecting the mitochondrial cytochrome-c oxidase demonstrated in this disorder [82], that is characterized by other considerable changes affecting the mitochondria (the neuronal ones in particular), such as the accumulation of osmiophilic material and the decreased size [84]. The previously mentioned accumulation of metal ions (particularly copper, iron and zinc) in the neuritic plaques is a critical stage favoring the progression of AD. In fact, since ions often act as co-factors with proteins, the reduction of their bioavailability (due to their accumulation) dramatically affects neuronal functioning and many enzymatic processes, including the degradation of Aβ. Moreover, ions produce free radicals, thus favoring OS. The latter damages the binding sites for ions, directly disturbing the interaction between ions, such as zinc, and proteins, with detrimental effects on neuronal signaling [85,86]. In addition, OS seems to be directly involved in the formation of the typical lesions of AD: senile plaques and NFTs. In fact, it interferes with the functioning of both endoplasmic reticulum (ER), fundamental for the regulation of protein folding, and beta-site APP cleaving enzyme 1, involved in the beta-secretase cleavage of the APP [78]. In addition, ROS directly determine structural changes in the proteins, favoring their misfolding, as occurs for Aβ. In particular, it has been demonstrated that the lipid oxidation product 4-hydroxy-2-nonenal modifies the three histidine residues in amyloid beta proteins, increasing their misfolding [87]. OS can also alter the metabolism of Aβ through the peroxidation of the ApoE. It has been demonstrated that the protective isoform of ApoE, the ε2 allele, is a powerful free radical scavenger, while ApoE ε4 is very sensitive to OS: lipid peroxidation occurs more easily in the presence of this allele [82]. In a vicious cycle, Aβ itself not only causes mitochondrial dysfunctions, thus favoring OS, but also produces ROS, particularly in the presence of other free radicals [84,82]. Despite Aβ possesses oxidative and hydrolytic properties, studies in vitro have highlighted its antioxidant activities. In fact, Aβ is a redox-metal chelator and its deposition could even represent a compensatory mechanism, aimed to isolate the highly reactive metals accumulated in the AD brain. The by-product of its antioxidant activity, such as hydrogen peroxide, could mediate the Aβ-related toxicity, accumulating in the brain once the amyloid deposition increases. Since the brain environment in vivo is very complex, the behavior of Aβ in vivo could be different, but these findings are nonetheless interesting, particularly if we think to the Aβ deposition occurring after a traumatic injury [88]. As far as tau is concerned, its misfolding and accumulation is favored by hyper-phosphorylation, stimulated by OS; the latter mediates the tau-related neurodegenerative processes through the enhancement of the tau-mediated cell cycle activation, that leads to neuronal death [89,90].

### Oxidative stress and neuroglia

Neurodegeneration is not only linked to neurons, but also to neuroglial cells. In fact, recent evidences suggest a pivotal role of neuroglia and OS-induced neuroglial alterations in AD. In fact, astrocytes in AD brains are characterized by OS-induced DNA damage [91]. The response to DNA double-stranded breaks includes the phosphorylation of the protein H2AX, belonging to the H2A histone family. High levels of this protein have been found in astrocytes localized in hippocampus and cerebral cortex, severely damaged in AD. The destiny of a cell activating this response is variable: if the damage cannot be repaired, apoptosis is likely to occur; alternatively, the cell could attempt to repair the damage until its resistance fails. It is possible that during chronic cytotoxic stress, as occurring in AD, the latter mechanism occurs. In any case, the presence of DNA damage indicates the presence of compromised astrocytes that could not be able to effectively support neurons as usual [92]. Oligodendrocytes, too, are involved in AD. In fact, the myelin breakdown has been associated to this type of dementia and could even precede the amyloid and tau pathology. On the other hand, Aβ and NFTs contribute to the oxidative damage to oligodendrocytes, further altering the myelination process [93]. Considering the ependymal cells, the OS-related damages affecting their carriers could affect the clearance of Aβ. For instance, P-glycoprotein and low-density lipoprotein receptor-related protein-1, two OS-sensitive proteins transporting Aβ, display functional impairments in AD [94-96]. The neural stem cells, too, are deeply affected by the oxidative status. Under normal conditions, proliferative neural stem cells need to maintain high levels of ROS; in fact, they are particularly resistant to the OS-related insults. However, the endogenous ROS levels must be finely regulated in order to avoid damages, also thanks to a rich pool of antioxidant defenses, probably activated when the cell is in a quiescent state [97]. In AD, neurogenesis is activated as a compensatory mechanism. However, OS damages the newly generated cells [98]. In addition, the failure of neurogenesis in crucial cerebral regions, such as hippocampal dentate gyrus, is strictly linked to the memory impairment [99]. Moreover, Aβ can directly affect the stem cells functioning. In fact, it has been demonstrated to cause morphological alterations of the NG2-positive cells, such as retraction of processes and dense cell bodies, that could further decrease the possibilities of repairing the already mentioned myelin breakdown [100,93]. Microglia, too, is strictly linked to OS, that represents a powerful microglial activator. The activation of microglia is the main determinant of neuroinflammation, another molecular basis of AD, characterized by the massive release of microglial pro-inflammatory cytokines (which expression is deeply altered in AD) and the formation of free radicals and oxidants, such as peroxynitrite. The latter is responsible for the nitration of tyrosines and nitrosylation of cysteines within enzymes and structural proteins and has been proposed to mediate the Aβ toxicity. In fact, amyloid peptides induce microglial activation, even if neuroinflammation can even preced amyloid and tau pathology [5,101]. OS and neuroinflammation are not mutually exclusive, but strictly intertwined: while OS induces microglial activation, vice versa neuroinflammation promotes the oxidative status [102]. More details on neuroinflammation in AD are reported below.

### Neuroinflammation

As previously discussed in the paragraph “functions of microglia”, microglial activation is a physiological response to various stimuli and aims to limit the damages. However, neurodegenerative disorders, such as AD, are characterized by chronic neuroinflammation that, in a self-perpetuating mechanism, determines the release of many pro-inflammatory cytokines, allows cellular infiltration in the brain through the disruption of the BBB and favors OS, thus creating the conditions for further microglial activation. The inflammatory status stresses the neurons leading them to their death, thus contributing to the pathogenesis and progression of AD [103]. Since neuroinflammation may begin before any significant neuronal loss, it could trigger the neurodegenerative process, for example in the presence of a genetic predisposition.

### Neuroinflammation in AD

When amyloid deposition occurs, microglia attempts to repair the damage. In vitro studies have highlighted that microglia can phagocytize Aβ and neurons exposed to it. However, in vivo studies have demonstrated that microglia cannot phagocytize Aβ in the presence of inflammatory cytokines. In fact, the neuroinflammatory condition occurring in vivo, inhibiting the potentially neuroprotective functions of microglia, could mediate AD progression favoring the uncontrolled accumulation of senile plaques [71]. As a matter of fact, the inflammatory status promotes microglial toxicity rather than neuroprotection [102]. On the other hand, neuroinflammation could directly promote amyloid deposition increasing the amyloidogenic processing of APP and/or interfering with the clearance of Aβ [102]. For example, interferon gamma, interleukin (IL) 4 and TNF-α have been demonstrated to promote Aβ deposition and reduce its clearance [104,105]. The importance of neuroinflammation is confirmed by the fact that genes encoding for immune receptors, such as TREM2 and CD33, have been associated to sporadic AD. Interestingly, TREM2 is highly expressed in microglial cells, being involved in the regulation of the phagocytic clearance of cellular debris [106]. Chronic neuroinflammation contributes to tau deposition, too, activating pathways leading to the formation of tau-insoluble fractions [71]. From a clinical point of view, neuroinflammation contributes to the occurrence of cognitive deficits. For example, IL-1 and pro-inflammatory prostaglandins have been proven to determine memory deficits [107]. However, it should be considered that not only inflammation is accompanied by anti-inflammatory mechanisms, but also the inflammatory pathways are extremely complex and dynamic. In this regard, IL-6, demonstrated to stimulate the synthesis of APP and exacerbate the Aβ-induced neuronal damage, can also exert positive effects suppressing Aβ deposition and stimulating microglia to phagocytize Aβ [108,109]. It is possible that the genetic polymorphisms of IL-6 could account for its flexible role, as well as a differential risk for AD relates to the genetic polymorphism of the anti-inflammatory IL-10 [110,111]. The serum levels of the latter are tipically elevated in AD and have been found to be inversely correlated to Aβ deposition in the CSF [112]. The anti-inflammatory peroxisome proliferator-activated receptor-γ is upregulated in AD, too, and contributes to limit the Aβ-related neuroinflammatory response [102].

### Neuroinflammation and neuroglia

Neuroinflammation is not only due to microglia, but also to the other glial cells, astrocytes in particular. For example, the exposure to the environmental toxicant named lead determines the activation of astrocytes, that release pro-inflammatory cytokines, produce more ROS and switch their metabolism trough the consumption of stored glycogen, thus depriving the neurons of their energy sources and contributing to the neuronal loss [113]. In AD, activated astrocytes accumulate near senile plaques, where they could interact with microglia through the release of regulatory molecules. In addition, astrocytes may be able to stimulate the migration of mature macrophages towards the inflammatory sites [114]. Similarly, microglia and oligodencrocytes engage in a close relationship with different outcomes: demyelination or myelin repair. Oligodendrocytes exposed to stress produce a variety of cytokines that stimulate microglia to migrate to the site of damage. According to the source of stress and the cytokine milieu, the interaction between oligodendrocytes and microglia could result in neuroprotection (e.g. recruitment of oligodendrocytes progenitors) or neurodegeneration (death of oligodendrocytes, demyelination) [31]. Ependymal cells, too, suffer from the effects of neuroinflammation, that threatens the integrity of the BBB and the BCB. For instance, the pro-inflammatory molecule nuclear factor kappa beta, released by activated astrocytes, determines structural changes in the ependyma, such as the loss of cilia. In fact, hydrocephalus and neuroinflammation are frequently associated [115]. The neural stem cells could potentially limit the detrimental effects of neuroinflammation through gliogenesis and neurogenesis; however, they fail to counterbalance the cellular loss. The sustained neuroinflammation may contribute to this failure, altering the ability of the neural stem cells to proliferate and differentiate. In fact, despite the fine regulation of the neural stem cells is not fully known, the inflammation-induced DNA methylation and histone modifications in the stem cells could at least account for an altered expression of neurotrophic factors, that are fundamental stimuli for the neural stem cells [116]. OS and neuroinflammation may sinergistically alter the neural stem cells functioning. In fact, not only OS contributes to the lack of trophic support, but also the neuroinflammation-related overproduction of ROS negatively affects neurogenesis [117,118].

This data confirms that the molecular bases of AD do not represent independent processes. In fact, neuroinflammation and OS are strictly linked to each other, but also with the other molecular basis of AD: the dysfunctional GSK-3 pathway, described below.

**Figure 1.**
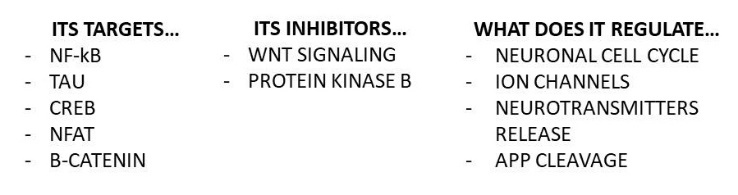
GSK3, a protein ubiquitously expressed in the brain. Some of the targets and inhibitors of GSK-3, as well as some fundamental brain processes regulated by this kinase. GSK-3: glycogen synthase kinase-3; NF-kB: nuclear factor KB; CREB: cAMP; NFAT: nuclear factor of activated T cells; APP: amyloid precursor protein.

### GSK-3 pathway

GSK-3 (Fig.1) is a serine/threonine kinase specific for glycogen synthase involved in various physiological processes, among which neurodevelopment, signaling and cell proliferation/death [119]. Two GSK-3 isoforms, α (51?kDa) and β (47?kDa), are known. A variant of GSK-3 β, GSK32, has been discovered in 2002. Many of the substrates of GSK-3 need a priming phosphate (a phosphorylated serine/threonine residue) triggering the phosphorylation process, usually determining the inactivation of the substrate. GSK-3 is highly represented in the brain and exerts its action on extremely important substrates, such as nuclear factor kappa beta, cAMP response element-binding protein, tau, nuclear factor of activated T cells, beta-catenin [119,120]. GSK-3 is constitutively active, but its activity is finely regulated through: a) the need of a priming phosphate; b) serine (inhibitory) and tyrosine (stimulatory) phosphorylation by other kinases; c) autophosphorylation; d) subcellular localization (regulation of transcription factors); d) activatory proteolytic cleavage. Protein kinase B and Wnt signaling pathway are two inhibitors of GSK-3 [119]. During brain development, the GSK-3 signaling regulates neurogenesis, neuronal polarization and axon growth [121]. In the mature brain, it regulates neuronal cell cycle, including neuronal death/survival, ion channels and neurotransmitters release, thus regulating the synaptic transmission [119]. A GSK-3 dysfunctional pathway, in terms of hyperactivity, has been claimed to be involved in the pathogenesis and progression of AD.

### Dysfunctional GSK-3 pathway in AD

The GSK-3 hypothesis of Alzheimer’s Disease, postulated by Hooper et al. in 2008, states that GSK-3 over-activity is involved in the amyloid cascade of AD, being responsible for an increased Aβ production and tau hyper-phosphorylation. It also favors the local responses to amyloid deposition and, stimulating apoptosis, it contributes to the neurodegenerative process [8]. More specifically, it has been demonstrated that GSK-3α regulates APP cleavage favoring the production of Aβ [122]. In addition, GSK-3 is responsible for the phosphorylation of tau. An over-activity of GSK3 in sporadic AD could be re-conducted to dysfunctional upstream signaling intermediates. For example, the risk for AD in APOE ε4 negative individuals is enhanced by the low-density lipoprotein receptor related Protein 6, a co-receptor for Wnt signaling, the latter inhibiting GSK-3 [8]. The Wnt signaling is involved in the inflammatory process and can drive both neurodegenerative and neuroprotective mechanisms. Interestingly, Wnt mediates the fine cross-talk between astrocytes and microglia during the inflammatory condition. For example, astrocytes are able to stimulate the Wnt signaling thus limiting the microglial inflammatory response also trough GSK-3 inhibition. However, the excessive microglial activation characterizing chronic neuroinflammation leads to a dysfunctional Wnt signaling and interferes with the anti-inflammatory mechanisms: as a result, the GSK-3 over-activity is further stimulated [123]. Notably, Aβ inhibits the Wnt signaling favoring the pro-inflammatory condition [124]. Interestingly, the genetic mutations in familial AD could affect GSK-3 activity, since for example presenilin 1 mediates the interaction between tau and GSK-3 and its mutations result in an enhanced tau hyper-phosphorylation [8, 125]. As previously stated, according to the amyloid cascade hypothesis, Aβ is responsible of the disease progression. In line with this theory, Aβ itself seems to be able to stimulate GSK-3, thus leading to tau phosphorylation [126]. In addition, GSK-3 impairs the cholinergic neurotransmission, crucially involved in memory. In mice, it has been demonstrated to disturb the cellular distribution of choline acetyltransferase, preventing long-term potentiation and negatively affecting spatial learning [127,8]. As to further confirm that the molecular bases of AD are intertwined, GSK-3 is sensitive to redox homeostasis, mediates oxidative damage, interferes with mitochondrial functioning and, being a regulator of the ER stress response, controls protein folding [119].

### Dysfunctional GSK-3 and neuroglia

GSK-3 can enhance neuroinflammation, also according to its cellular localization. For instance, it has been demonstrated that GSK-3 stimulates the production of TNF-α by activated microglia and controls microglial migration, thus contributing to the inflammation-induced neurotoxicity [119,7,128]. GSK-3 also interacts with the other glial cells, favoring AD progression. In fact, GSK-3 reduces the tolerance of astrocytes against the inflammatory damage and inhibits oligodendrocytes differentiation and myelination. As a result, the neuroprotective mechanisms that could potentially avoid further cellular loss are compromised [129,6]. Moreover, the complex pathway involving Wnt, beta-catenin and GSK-3 seems to be involved in the disruption of the BBB occurring in AD, via the altered expression of multidrug efflux transporters and P-glycoprotein by the ependymal cells [130-132]. Neural stem cells are not exempt from GSK-3 control; in fact, this kinase is involved in the complex regulation of their differentiation, proliferation and self-renewal through divergent pathways, depending on the tissue of origin [133]. Summarizing, even when the dysfunctional GSK-3 pathway is considered, the involvement of neuroglial cells in AD is confirmed.

### Glial changes and neurodegeneration in AD: underlying mechanisms

**Figure 2.**
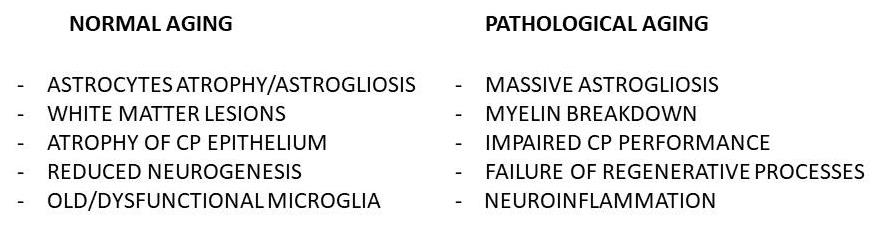
Glial cells in normal and pathological brain aging. The figure summarizes the main glial changes occurring in normal versus pathological brain aging. CP: choroid plexus

As described before, neurons and glial cells are deeply involved in the path towards neurodegeneration. However, the mechanisms underlying the latter are very complex and still debated. A crucial step seems to be the synaptic damage, followed by neuronal loss [134]. Amyloid pathology leads to synaptic dysfunctions and glial alterations through different mechanisms, among which disruption of Ca2+ signaling, activation of immunoproteasome, induction of glutamate neurotoxicity, mitochondrial dysfunction and alteration of the signaling pathways involved in synaptic plasticity and neurogenesis [134-137]. Brain aging implies the occurrence of glial changes, that appear to be more dramatic in AD (Fig. 2), thus contributing to AD pathogenesis and progression, as described below. The mechanisms underlying glial changes in AD and the significance of these changes in terms of neurodegeneration are reported below.

### Astrocytes in AD

In animal models of AD, the neuritic plaques are surrounded by astrogliosis, consisting in the presence of hypertrophic, reactive astrocytes expressing high levels of glial fibrillary acidic protein (GFAP), whereas astroglial atrophy occurs throughout the brain parenchima. In fact, astrocytic reactivity and GFAP expression show regional differences. For instance, astrogliosis is more represented in the hippocampus, while the relative deficit in reactivity of the prefrontal cortex reflects its vulnerability to AD lesions, being astrogliosis a defensive mechanism. Unfortunately, the massive and sustained astrogliosis occurring in AD contributes to the anatomical devastation of the brain [138, 139]. The regional differences in astrocytic reactivity account also for the co-presence of atrophic and reactive astrocytes in AD brains: in general, astrocytes near the plaques undergo astrogliosis and contribute to the glia scar, whereas astrocytes distal to the damage undergo atrophy. While astrocytes atrophy could contribute to the early synaptic damage, astrogliosis could exacerbate the damage thus favoring AD progression. Not by chance, atrophy and synaptic loss can be detected in the very initial stages of AD, when the presence of plaques is rather limited [140, 138]. According to this evidence, astrocytes could be considered as the first Aβ target as well as mediators of its neuronal toxicity [141,142]. The Aβ-induced astrogliosis seems to involve redox mechanisms and is characterized by fundamental changes in the astrocytic functioning, affecting Ca^2+^ signaling (fundamental for the neuron-astrocyte crosstalk), gliotransmitters production, neurovascular unit functioning, mitochondrial performance (resulting in ROS overproduction) and antioxidant defenses. Due to the neuronal dependence on astrocytes, these changes threaten neuronal survival, thus mediating AD progression [141-144]. Another aspect disrupting astrocytes functioning in AD is represented by tau toxicity, mostly related to tau aggregation, cytoskeletal perturbations and altered gene transcription. Its functional consequences interfere with important astrocytic functions, such as glutamate transport and regulation of the BBB [145]. In addition, the protein astrogliopathy, frequently reported in AD, leads to the lysis of Aβ-burdened astrocytes and contributes to the amyloid plaques formation [146,147].

### Oligodendrocytes in AD

Similarly to astrocytes, oligodendrocytes seem to play an active role in AD progression. According to a development-to-degeneration model, the protracted myelination process of the cortical areas could be the key for their vulnerability to neurodegeneration. More specifically, the metabolic demands of the myelination process account for the oligodendrocytes vulnerability to brain insults while creating the ideal environment for pathological brain aging (e.g. increase of intracortical cholesterol and iron) in those areas characterized by protracted myelination [148]. The oligodendrocytes susceptibility to stressors could explain the myelin breakdown typical of older ages, while the response aimed to restore myelin could cause further damage to oligodendrocytes and favor the deposition of AD lesions, and the consequent neuronal loss, in the most vulnerable brain areas [148]. In fact, even if the myelin breakdown was a secondary feature in AD (e.g. consequential to the neuronal loss), the progressive disruption of the neuronal circuits would certainly represent a substantial contributor to the disease progression [148, 149]. On the other hand, white matter lesions (subcortical degeneration and ischaemic-hypoxic changes) are largely related to glial changes and can be detected in the very early stages of AD, preceding sometimes amyloid and tau pathology [150, 151]. Moreover, oligodendrocytes directly endure tau and amyloid toxicity [145,149]. As for astrocytes, tau pathology affects oligodendrocytes mostly through tau aggregation, cytoskeletal perturbations and altered gene transcription. Being rich in microtubules, oligodendrocytes probably need tau more than the other glial cells. Early axonal contact, microtubules stabilization and myelination involve oligodendrocytic tau. As a result, the tau lesions severely affect the myelination process as well as the axonal transport [145]. As far as amyloid pathology is considered, oligodendrocytes are highly susceptible to OS-related Aβ toxicity, that could break their fragile homeostasis and induce their death, also through the alteration of the oligodendrocytic apoptosis signaling [149, 150, 152, 153]. These data have undermined the initial concept of AD as a disease primarily affecting grey matter. Glial changes are important determinants of the white matter lesions, as confirmed by neuropathological evidence relating the severity of fronto-parietal lesions to the astrocytes/oligodendrocytes ratio in human AD brains [154].

### Ependymal cells in AD

Along with capillary reabsorption and enzymatic catabolism, the CSF transport is another mean of Aβ monomers clearance. Hence, an impaired CSF dynamic favors oligomerization, thus contributing to plaques formation [155]. At older ages, the CP epithelium and ependymal cells undergo atrophic processes that, probably through immunological mechanisms, become particularly severe in AD. As a result, the performance of the CP (characterized by epithelial atrophy, thickening of the basement membrane and stroma fibrosis) is dramatically impaired and the insufficient Aβ clearance could favor AD progression [156]. This hypothesis is supported by the demonstrated correlation between degenerative processes of the ependymal cells and plexus (including the presence of Biondi inclusions, that is thread- and tangle-like inclusions in the CP) and those affecting the cerebral cortex [157]. In addition, the ependymal cells of the AD brain display an altered expression of aquaporin 4 and megalin; interestingly, the severity of these alterations relates to the number of senile plaques [158, 159]. On the other hand, the ependymal layer is rich in advanced glycation end-product receptors (RAGE), responsible for the influx transport of glycated Aβ from the blood to the brain [160]. Consequently, an increased expression of RAGE could progressively sustain amyloid pathology, being present from the early to the advanced stages of AD [161]. From a research performed on rodents, the blockade of the insulin-like growth factor I receptor in the CP resulted in brain amyloidosis, cognitive disturbances, hyper-phosphorylated tau deposits, gliosis and synaptic protein loss [162]. However, the role of CP is not limited to the Aβ clearing. In fact, it releases neurotrophic factors and contains a subpopulation of multipotent cells, thus showing a neurogenic potential [163]. However, despite Aβ exposure stimulates neurogenesis in the CP, it decreases at the same time the survival of the newly born neurons [164].

### Brain stem cells in AD

Neurogenesis and gliogenesis are two interrelated processes, since the brain stem cells can often differentiate into both neural and glial lineage [165]. The value of these neuro-regenerative processes is still debated, but they could play an important role in brain plasticity, favoring the learning processes and the memory formation throughout life, despite both neurogenesis and maturation of newly born neurons decrease with age [166, 167]. The role of neurogenesis and gliogenesis and their Aβ-induced modifications in neurodegeneration is still debated [168-170], but many studies have demonstrated an impaired neurogenesis in AD [171,172], that could be more pronounced in the tardive stages and senile forms of AD [169, 170] and account at least in part for the cognitive dysfunctions [172]. The amyloid pathology seems to negatively interfere with the functioning of the brain stem cells through several mechanisms, among which altered cellular signaling [172, 173], induction of OS and neuroinflammation [174, 175], downregulation of transcription factors [176], apoptosis promotion in the neural lineage, altered calcium homeostasis [177], telomere attrition, attenuated motility [178], altered morphology and proteoglycan expression in NG2-positive cells [100]. In addition, APP and APP-derived fragments could directly reduce neurogenesis independently from Aβ [179]. However, the neurogenic response in AD is very complex and could be variable according to the AD form and stage. For instance, if Aβ could reduce neurogenesis while favoring gliogenesis (microglial proliferation in particular) from the early disease stages [168], other studies report an increase in neurogenesis at the Aβ plaque free stage and a decrease in the later stages [169] or an increased hippocampal neurogenesis that continues during disease progression [180]. It would not be strange if an increase of neurogenesis and gliogenesis existed in AD but, unfortunately, this compensatory attempt seems to fail, also considering the functional alterations occurring in AD and creating a hostile brain environment that would not allow a long survival of the newly born cells, thus preventing their maturation [98, 176, 181]. Another factor disturbing neurogenesis efficiency in AD is represented by tau pathology. Since adult neurogenesis requires an efficient microtubule network [182], tau protein is obviously involved in synaptic plasticity and even in the regulation of death and survival of immature neurons [183], so that it could mediate the failure of hippocampal neurogenesis in conditions of chronic stress [184]. In the light of these data, improving the knowledge on the neurogenic and gliogenic mechanisms in AD becomes fundamental. In fact, if on the one hand a dysfunctional neurogenesis could be involved in the disease progression, on the other hand efficient neuro-regenerative processes could compensate the massive cellular loss occurring in AD, therefore showing a therapeutic potential [185].

### Microglia in AD

The role of microglial-related neuroinflammation as a cause or consequence of neurodegeneration is constantly debated. However, many evidences suggest a central role of neuroinflammation in tau and amyloid deposition [71, 102] as well as in glial alterations [31, 115]. In addition, neuroinflammation could trigger the neurodegenerative process [186]. In fact, it is an early feature of AD [187] and the presence of activated microglia near the plaques has been widely reported, characterizing the disease from the initial to the advanced stages, when it eventually wanes [188]. Moreover, since microglial cells display different reactive states, they could exert different roles throughout the natural history of the disease [189]. A recent research has reported that the hypoxic damage, known to exacerbate amyloid and tau pathology, could be involved in neuroinflammation through the induction of the M1 (pro-inflammatory) microglial phenotype at the expenses of the M2 (protective) one [190]. In addition, AD is characterized by an unbalance between inflammatory and clearing properties of microglia [189] and it has been proposed that lowering the Aβ burden would restore an efficient microglial functioning [191]. Notably, microglial cells could directly contribute to the neuronal loss via different mechanisms, such as Aβ-induced phagocytosis [192] and excessive synaptic pruning performed synergistically with complement-dependent pathways [193]. More specifically, not only microglial cells express complement receptors, but they probably represent the major source of C1q (the initiating protein of the classical complement cascade) in the brain, as inferred from a study performing cell-specific deletions of C1qa. Physiologically, C1q activates C3 which, in turn, opsonizes the synapses to be recognized and eliminated by microglial cells via the stimulation of the microglial complement receptor 3 [193, 194]. The downstream factors C5, C6, C7, C8 and C9 synergistically create the so-called membrane attack complex responsible for the cell lysis [195]. In AD, the presence of Aβ oligomers determines an aberrant activation of the complement cascade, even before plaque deposition [193]. In APP/PS1 C3 knockout mice, the glial reaction to plaque deposition is attenuated. As a result, these mice are protected by synaptic loss and display better cognitive performances despite their plaque load [196]. On the other hand, C3 can favor Aβ clearance and its deficiency has been related to accelerated plaque deposition as well as loss of specific subsets of neurons [197]. A possible explanation of these inconsistent findings could lay in the fact that the modulation of the complement cascade is altered in AD so that the negative effects of complement factors could relate to qualitative, more than quantitative, aspects. Evidence reporting that AD patients show increased levels of C9, a final component forming the membrane attack complex, as well as a deficiency of the complement defense protein CD59 (inhibiting the cell lysis), further highlight the lack of regulatory mechanisms of complement-mediated processes in AD. In fact, the exposure to Aβ determines the downregulation of protective molecules while activating microglia, thus leading to neuronal loss [195]. The latter was interestingly prevented by the elimination of microglia in aggressive models of amyloid pathology [198]. Moreover, neuronal loss, along with microglial activation, preceded the formation of NFTs in a mouse model of tauopathy [199]. In addition, in a triple transgenic model of AD the increase of resting microglia preceded both microglial activation and plaque formation, as if the brain was arming himself in preparation of the profound distorsions to come [200]. Despite these data, the link between neuroinflammation and neurodegeneration has been questioned by another hypothesis. More specifically, since microglial cells undergo replicative senescence, the presence of old and/or dysfunctional microglial cells could represent the crucial step towards neurodegeneration [201]. The histo-pathological findings obtained from humans affected by none to severe AD pathology/Down’s syndrome support this hypothesis, demonstrating that dystrophic (senescent), rather than activated microglial cells, were co-localized with the AD lesions. In this study, microglial degeneration was found to progress along with neurodegeneration, so that in those cases characterized by advanced neurofibrillary and amyloid pathology the microglial cells appeared to be completely destroyed. Hence, the major contributors to neurodegeneration in sporadic AD would not be activated microglial cells, but dystrophic ones, unable to provide neuroprotection [202].

### Conclusions

In the light of what has been since here discussed, it is apparent that neuroglia plays a fundamental role in both physiology and pathology of the brain. In fact, the main molecular bases of AD, namely OS, neuroinflammation and dysfunctional GSK-3 pathway share neuroglia as a co-determinant and/or target. Since the molecular alterations occurring in AD are intertwined, manipulating one molecular process could have reverberating effects on the other. The glial changes occurring from the early to the advanced disease stages could considerably contribute to the neurodegenerative process. However, the role of neuroglia in neurodegeneration is still broadly debated. Further studies are needed to shed light on such a complex as well as fascinating topic.
